# Resilience and its associations in children with Systemic Lupus Erythematosus and Juvenile Idiopathic Arthritis

**DOI:** 10.1186/s12969-023-00854-3

**Published:** 2023-07-07

**Authors:** Rebecca Trachtman, Julie Samuels, Emma Wojtal, Brian M Feldman

**Affiliations:** 1 Icahn School of Medicine at Mount Sinai, 1 Gustave Levy Place, 10029 New York, NY USA; 2 Mindich Child Health and Development Institute, New York, NY USA; 3 The Hospital for Sick Children, Toronto, ON Canada; 4 University of Toronto, Toronto, ON Canada

**Keywords:** Resilience, Health-related quality of life, Patient-reported outcomes, Juvenile idiopathic arthritis, Systemic lupus erythematosus

## Abstract

**Background:**

Resilience has been shown to be associated with better psychological outcomes and ability to cope with negative and traumatic events in the healthcare setting. Therefore, in this study, we aimed to evaluate resilience and its association with disease activity and health-related quality of life (HRQOL) in children with Systemic Lupus Erythematosus (SLE) and Juvenile Idiopathic Arthritis (JIA).

**Findings:**

Patients with diagnoses of SLE or JIA were recruited. We collected: demographic data, medical history and physical examination, physician and patient global health assessments, Patient Reported Outcome Measurement Information System questionnaires, Connor Davidson Resilience Scale 10 (CD-RISC 10), Systemic Lupus Erythematosus Disease Activity Index, and clinical Juvenile Arthritis Disease Activity Score 10. Descriptive statistics were calculated, and PROMIS raw scores were converted to T-scores. Spearman’s correlations were performed, with statistical significance set to p < 0.05. 47 study subjects were recruited. The average CD-RISC 10 score in SLE was 24.4, and in JIA was 25.2. In children with SLE, CD-RISC 10 was correlated with disease activity and inversely correlated with anxiety. In children with JIA, resilience was inversely associated with fatigue, and positively correlated with mobility and peer relationships.

**Conclusions:**

In children with SLE and JIA, resilience is lower than in the general population. Further, our results suggest that interventions to increase resilience may improve the HRQOL of children with rheumatic disease. Ongoing study of the importance of resilience in this population, as well as interventions to increase resilience, will be an important area of future research in children with SLE and JIA.

**Supplementary Information:**

The online version contains supplementary material available at 10.1186/s12969-023-00854-3.

## Introduction

The importance of resilience, defined as the capacity to withstand or to recover quickly from difficulties, is increasingly recognized in the healthcare setting. Prior research has shown that individuals with high resilience have better psychological outcomes and are able to cope better with negative and traumatic events [1]. In contrast, adversity and psychological stress can lead to or exacerbate chronic illness in children [2, 3]. Further, research evaluating resilience strategies in pediatric oncology patients revealed that interventions to increase resilience led to better cancer-specific quality of life scores and lower psychological distress [4].

Children with a variety of severe chronic inflammatory diseases are cared for by pediatric rheumatologists, typically requiring long-term treatment and frequent monitoring. Previous work in children with chronic disease has shown that higher resilience is associated with greater transition readiness [5]. Additionally, one study indicated that children with non-inflammatory chronic musculoskeletal pain had lower resilience levels than healthy children and children with chronic medical conditions [6].

Systemic Lupus Erythematous (SLE) and Juvenile Idiopathic Arthritis (JIA) are two of the most common systemic inflammatory diseases seen in the pediatric rheumatology outpatient setting. Data in adults with SLE reveals that higher resilience is linked to improved medication adherence [7, 8]. Further, one study showed that increased psychological flexibility, a component of resilience, in children with JIA and their parents helps reduce pain intensity [9]. However, there is a paucity of data about resilience in children with SLE and JIA.

Given all of this, in this study we aimed to evaluate resilience and its association with disease activity and health-related quality of life (HRQOL) in children with SLE and JIA.

## Patients and methods

Patients with diagnoses of SLE or JIA, based on Systemic Lupus Erythematosus International Collaborating Clinics Groups and International League of Associations for Rheumatology criteria, respectively, were consecutively recruited between 8/2019 and 12/2022 in a pediatric rheumatology outpatient setting [10, 11]. Patients with SLE included patients with or without lupus nephritis between 10 and 22 years old. SLE patients undergoing renal transplant or with active infection were excluded. Patients with JIA were between 1 and 22 years old; those with other rheumatologic disease or active infection were excluded. This study was approved by the Icahn School of Medicine at Mount Sinai Institutional Review Board.

Data collected at a single study visit included demographic data, medical history and physical examination, physician global health assessment (PhGA), patient global health assessment (PtGA), Patient Reported Outcome Measurement Information System (PROMIS) questionnaires, a measure of HRQOL (see below), and Connor Davidson Resilience Scale 10 (CD-RISC 10), a validated measure of resilience [12, 13]. The CD-RISC 10 contains 10 questions regarding resilience, and has a score of 0–40, with higher scores representing higher resilience. This validated measure has different means and standard deviations for various populations in which it has been tested. PROMIS domains were collected as computer adaptive tests (CATS) and comprised the anxiety, depression, fatigue, mobility, pain interference, peer relationships, and physical activity domains. When converted to T-scores, PROMIS CATs have a range of 0-100, with 50 representing the mean, scores higher than 50 representing more of the domain being measured, and scores below 50 representing less of the domain being measured. Importantly, patients completed surveys when aged 8 or above; caregivers completed surveys for study subjects less than 8 years of age.

The treating physician completed the physician global health assessment (PhGA), and the Systemic Lupus Erythematosus Disease Activity Index (SLEDAI), a validated measure of disease activity in SLE, was calculated for patients with SLE [14]. The SLEDAI ranges 0-105, with 0 representing no activity, 1–10 indicating mild/moderate activity, and scores greater than 10 indicating high disease activity. The clinical Juvenile Arthritis Disease Activity Score 10 (JADAS), a validated measure of disease activity in JIA, was calculated for patients with JIA. This score ranges 0–40, with higher numbers indicating higher disease activity [15].

### Statistics

Description statistics were calculated. Using standard software, raw scores for PROMIS measures were converted to T-scores. Mann-Whitney U test and Spearman’s correlations were performed as appropriate, with statistical significance set to p < 0.05.

## Results

### SLE

21 children were enrolled in this study (4 declined participation – Table 1). Study subjects had an average age of 15.1 years, and the majority of patients were female (90.5%) and had Medicaid insurance (85.7%). 38.1% of patients reported their disease duration was between 0 and 6 months, and the average SLEDAI score of 8.1 is consistent with moderate disease activity. The following medications were reported: hydroxychloroquine (15/21, 71%); prednisone (13/21, 62%); mycophenolate mofetil (9/21, 43%); ACE inhibitors (3/21, 14%); and rituximab (2/21, 10%). The average CD-RISC 10 score was 24.4, substantially lower than the average score for the general population.

**Table 1:** Demographic Characteristics

	Characteristic	SLE	JIA
		n = 21	n = 26
Age (years, average ± SD)		15.1 ± 2.6	11.7 ± 5.0
Gender			
	Female	19 (90.5%)	17 (65.4%)
	Male	2 (9.5%)	9 (34.6%)
Race			
	Asian	2 (9.5%)	4 (15.4%)
	Black or African American	8 (38.1%)	5 (19.2%)
	White	3 (14.3%)	6 (23.1%)
	Unknown/Not Reported	8 (38.1%)	11 (42.3%)
Ethnicity			
	Hispanic or Latino	14 (66.7%)	17 (65.4%)
	Not Hispanic or Latino	6 (28.6%)	8 (30.8%)
	Unknown/Not Reported	1 (4.8%)	1 (3.8%)
Insurance			
	Private	2 (9.5%)	6 (23.1%)
	Medicaid	18 (85.7%)	20 (76.9%)
	Other	1 (4.8%)	0 (0%)
Disease Duration			
	0–6 months	8 (38.1%)	8 (30.8%)
	6–12 months	2 (9.5%)	8 (30.8%)
	12–18 months	0 (0%)	2 (7.7%)
	18–24 months	2 (9.5%)	1 (3.8%)
	2–3 years	3 (14.3%)	4 (15.4%)
	> 3 years	6 (28.6%)	3 (11.5%)
JIA Subtype			
	Oligoarticular		3 (11.5%)
	Polyarticular		10 (38.5%)
	Psoriatic arthritis		2 (7.7%)
	Enthesitis-related arthritis		4 (15.4%)
	Systemic		5 (19.2%)
	Undifferentiated		2 (7.7%)
CD-RISC Score (average ± SD)		24.4 ± 9.1	25.2 ± 7.2
SLEDAI score (average ± SD)		8.10 ± 7.67	
JADAS score (average ± SD)			8.03 ± 9.43

As shown in Table 2, resilience was moderately correlated with the PROMIS anxiety measure (r = -0.50, p = 0.03), with greater resilience associated with lower anxiety; however, resilience was not correlated with any other PROMIS domains (p > 0.5). CD-RISC also moderately correlated with SLEDAI (r = 0.51, p = 0.02), with greater resilience associated with greater disease activity. CD-RISC was not correlated with age, PtGA or PhGA, or disease duration.

**Table 2:** Spearman Correlation Coefficients

		SLE CD-RISC 10 (r-value)	JIA CD-RISC 10 (r-value)
Age		-0.20	-0.01
PROMIS Domain			
	Anxiety	**-0.50**	-0.19
	Depression	-0.31	-0.06
	Fatigue	-0.37	**-0.51**
	Mobility	0.17	**0.41**
	Pain Interference	-0.45	0.19
	Peer Relationships	0.08	**0.45**
	Physical Activity	0.25	0.20
Physician Global Health Assessment		0.39	-0.06
Patient Global Health Assessment		0.19	-0.23
SLEDAI		**0.52**	
JADAS			-0.19

*bolded p < 0.05

### JIA

26 children were enrolled (3 declined participation). Study subjects had an average age of 11.7 years, and the majority of patients were female (65.4%) and had Medicaid insurance (76.9%; Table 1). JIA subtype included polyarticular (38.5%), systemic (19.2%), enthesitis-related (15.4%), oligoarticular (11.5%), psoriatic (7.7%) and undifferentiated (7.7%). Most patients reported a disease duration of under 1 year (61.8%), and average JADAS score was 8, indicating mild/moderate disease activity. The following medications were reported: non-steroidal anti-inflammatory drugs (10/26, 38%); methotrexate (5/26, 19%); adalimumab (6/26, 23%); interleukin-1 blockade (3/26, 12%); interleukin-6 blockade (1/26, 4%); and prednisone (2/26, 8%).The average CD-RISC 10 score was 25.2, lower than the average for the general population [13].

CD-RISC 10 was inversely associated with the PROMIS Fatigue domain (r = -0.51, p = 0.008) and positively correlated with Mobility (r = 0.41, p = 0.04) and Peer Relationship domains (r = 0.45, p = 0.02), indicating that greater resilience is associated with less fatigue, better mobility, and better peer relationships. CD-RISC 10 was not associated with other PROMIS domains, PtGA, PhGA, disease duration, or the JADAS score. The association of the CD-RISC 10 with the JADAS score was unchanged with and without the PtGA.

## Discussion

While prior studies have investigated resilience in adults with rheumatic disease and in children with chronic non-inflammatory musculoskeletal pain, ours is the first to assess resilience in children with SLE and JIA.

We found that, in general in children with SLE and JIA, resilience is lower than the general population; the average score for young adults/students in the United States ranges from 27.2 to 33.5 [13]. Further, we found that resilience in our population was lower than in adolescents and young adults with cancer, where there was consistently a mean of 29 and standard deviation of 6 [16, 17]. Resilience in children with SLE and JIA was similar to that in children with chronic musculoskeletal pain (mean 25.3) [6]. This highlights the possibility that musculoskeletal pain impacts resilience levels in a unique way, though the precise reasons for this remain unclear.

In SLE, lower resilience was correlated with higher anxiety levels, and interestingly, higher resilience was associated with higher disease activity. Perhaps this is because higher disease activity requires a greater amount of resilience; however, this highlights an area for future study.

In JIA, higher resilience was associated with decreased fatigue, increased mobility, and greater peer relationships; however, there was no correlation found with disease activity. This suggests that interventions to increase resilience may have the potential to improve the HRQOL of children with rheumatic disease.

Strengths of our study are that we evaluated resilience in a population of children with SLE and JIA and compared this to a range of pediatric and parent proxy PROMIS CATs, as well as to the standard disease activity measures in SLE and JIA, and to the most commonly used generic PRO. We also showed that evaluating resilience in our population can be done quickly and with ease.

Limitations of this study include a relatively small sample size, due to recruitment at a single center with limited clinical practice, with a limited sociodemographic population and low disease activity. It is important for future studies of resilience to assess larger, more diverse populations. Our findings should also be further explored in prospective studies to assess the relationship between resilience, HRQOL, and disease activity.

Resilience is a factor that has the potential to play a key role in the HRQOL of children with rheumatic disease. Ongoing study of the importance of resilience in this population, as well as interventions to increase resilience, will be an important area of future research in children with SLE and JIA.

## Electronic supplementary material

Below is the link to the electronic supplementary material.

Supplementary Material 1

## Data Availability

The datasets used and/or analyzed during the current study are available from the corresponding author on reasonable request.

## List of abbreviations

SLE: Systemic Lupus Erythematous
JIA: Juvenile Idiopathic Arthritis
HRQOL: Health-related quality of life
PhGA: Physician global health assessment
PtGA: Patient global health assessment
PROMIS: Patient Reported Outcome Measurement Information System
CD-RISC 10: Connor Davidson Resilience Scale 10
CATS: Computer adaptive tests
SLEDAI: Systemic Lupus Erythematosus Disease Activity Index
JADAS: Clinical Juvenile Arthritis Disease Activity Score 10
