# Supplementary material for: Resilience and its associations in children with Systemic Lupus Erythematosus and Juvenile Idiopathic Arthritis
Source: Pediatr Rheumatol Online J. 2023 Jul 7;21:67. doi: 10.1186/s12969-023-00854-3 (PMC10327309; doi:10.1186/s12969-023-00854-3)
Supplement: Supplementary file 1 — Supplementary Material 1 [file 12969_2023_854_MOESM1_ESM.docx]

**Resilience and its Associations in Children with Systemic Lupus Erythematosus and Juvenile Idiopathic Arthritis**

Rebecca Trachtman
Icahn School of Medicine at Mount Sinai
1 Gustave Levy Place, New York, NY 10029

06/15/2023

Dear Pediatric Rheumatology Editorial Board,

We wish to submit a revision of a Short report entitled “Resilience and its Associations in Children with Systemic Lupus Erythematosus and Juvenile Idiopathic Arthritis” for consideration by Pediatric Rheumatology.

We confirm that the manuscript has not been submitted or published elsewhere with the exception of abstracts published with scientific meetings.

Please address all correspondence concerning this manuscript to me:

Rebecca Trachtman

1 Gustave Levy Place, New York, NY 10029

Email: [rebecca.trachtman@mssm.edu](mailto:rebecca.trachtman@mssm.edu) Telephone: 212-241-1217

Thank you for your consideration of this manuscript.

Sincerely,

Rebecca Trachtman
